# Humidity‐Induced Degradation Mapping of Pt_3_Co ORR Catalyst for PEFC by In‐Operando Electrochemistry and Ex Situ SAXS

**DOI:** 10.1002/smll.202407591

**Published:** 2024-10-16

**Authors:** Joel Mata Edjokola, Marco Bogar, Maximillian Grandi, Rodolfo Taccani, Heinz Amenitsch, Marjan Marinšek, Viktor Hacker, Merit Bodner

**Affiliations:** ^1^ Institute of Chemical Engineering and Environmental Technology Graz University of Technology Graz 8010 Austria; ^2^ Department of Engineering and Architecture University of Trieste Trieste 34127 Italy; ^3^ Institute of Inorganic Chemistry Graz University of Technology Graz 8010 Austria; ^4^ Faculty of Chemistry and Chemical Technology University of Ljubljana Ljubljana 1000 Slovenia

**Keywords:** degradation, ORR electrocatalyst, polymer electrolyte fuel cells, relative humidity

## Abstract

Understanding the degradation mechanisms of Pt‐alloy catalysts is crucial for enhancing their durability. This study investigates the impact of relative humidity on Pt and Pt_3_Co catalysts using potential‐cycling‐based accelerated stress tests. Two conditions are investigated: 100% relative humidity on both sides, and a gradient with 30% at the anode and over 100% at the cathode. Pt_3_Co demonstrates sensitivity, with 77% performance loss and reductions in electrocatalyst surface area. Results demonstrate a 30% decrease in potential loss for Pt catalysts and a 77% increase for Pt_3_Co catalysts, indicating significant performance degradation in high humidity conditions, with Pt_3_Co exhibiting greater sensitivity. Measurements of electrochemically active surface area reinforce these findings. Resistance analysis using electrochemical impedance spectroscopy using equivalent circuit modeling reveals a threefold increase in Pt_3_Co MEAs' cathode charge transfer resistance and mass transport resistance during accelerated stress tests. Local current distribution analysis highlights differences between Pt catalyst and Pt_3_Co, with the latter displaying dealloying effects. Small‐angle X‐ray scattering reveals changes in particle and cluster sizes, indicating structural changes. Scanning electron microscopy highlights catalyst and membrane thickness variations, suggesting heterogeneity in Pt_3_Co. Under humidity gradients, Ostwald ripening plays a significant role in altering the catalyst's Pt_3_Co structure and subsequently impacting its performance.

## Graphical abstract

This study investigates the degradation mechanisms of Pt_3_Co catalysts under varying humidity conditions using accelerated stress testing. Pt_3_Co experiences a 77% performance loss, significant structural changes, and increased resistance, while Pt shows a 30% loss. Electrochemical impedance spectroscopy and small‐angle X‐ray scattering reveal Pt_3_Co's high sensitivity to humidity gradients, highlighting its vulnerability to dealloying and accelerated performance decay.

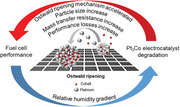

## 1 Introduction

Polymer electrolyte fuel cells (PEFCs) have gained popularity for their potential to revolutionize energy production and reduce our reliance on fossil fuels. Platinum (Pt), usually supported on a carbon substrate, is the preferred oxygen reduction reaction (ORR) catalyst. With the aim of extending catalyst stability over time, a vast effort has been made to understand Pt catalyst degradation in PEFCs, both from a mechanistic and an application‐focused point of view.^[^ ^1^, ^2^ ^]^ Nonetheless, the reliance on Pt has significant drawbacks due to its scarcity and high cost.^[^ ^3^ ^]^ The need to reduce catalyst costs without compromising system durability is the rationale behind focusing on Pt‐alloy made with transition metals like copper (Cu),^[^ ^4^ ^]^ iron (Fe),^[^ ^5^ ^]^ nickel (Ni),^[^ ^6^, ^7^ ^]^ and Cobalt (Co),^[^ ^8^, ^9^ ^]^ as a cost‐effective alternative to pure Pt catalysts. Pt alloys show interesting catalytic activities and increased functionality with reduced Pt content and costs. The observed improvement in ORR activity for these Pt‐alloys is thought to stem from alterations in the atomic structure of the alloy catalyst surface.^[^ ^10^, ^11^, ^12^, ^13^ ^]^ Min et al. noted a decrease in the Pt‐Pt distance on the alloy surface, which facilitates oxygen adsorption.^[^ ^14^, ^15^ ^]^

By harnessing the unique properties of these transition metals, many works aimed to strike a harmonious balance between catalytic activity and cost‐effectiveness, thereby making PEFCs competitive in the ever‐evolving energy market.^[^ ^16^ ^]^ In this framework, Pt_3_Co catalysts have emerged as a promising solution, showing remarkable performance in catalyzing the ORR, surpassing the capabilities of traditional Pt catalysts.^[^ ^9^, ^17^ ^]^ This breakthrough opened new possibilities for developing PEFCs with improved efficiency and reduced production costs. However, this new class of catalysts shows a different degradation behavior.^[^ ^18^ ^]^ In particular, in comparison with purely Pt catalysts, Co leaching was observed to induce a coarsening of the catalyst particle surface and to form a Pt‐rich shell structure on the outside.^[^ ^8^ ^]^ This process can also modify the catalyst structure and increase the surface area by creating a protective shell.

To improve catalyst design and develop effective degradation mitigation strategies, a promising approach consists in gaining new insights about catalyst degradation from both functional and structural points of view, *in operando* conditions. In fact, being catalyst degradation in fuel cells a complex phenomenon, different techniques need to be combined in order to gain a more complete picture about. Among the most common electrochemical techniques, polarization resistance characterization through electrochemical impedance spectroscopy (EIS)^[^ ^11^, ^12^, ^13^ ^]^ and Electrochemically active surface area (ECSA) calculation from cyclic voltammetry (CV),^[^ ^19^, ^20^ ^]^ are extensively used. In parallel, X‐rays‐based investigation techniques become in the last decades a valuable tool for monitoring catalyst degradation, helping in gaining important about either change of chemical environment, visa X‐ray absorption spectroscopy (XAS),^[^ ^21^, ^22^, ^23^ ^]^ either about catalyst morphological evolution, by means of small angle X‐ray scattering (SAXS).^[^ ^24^ ^]^ Recently, SAXS analysis conducted on both catalyst model systems,^[^ ^6^ ^]^ as well as on half‐cell electrodes,^[^ ^25^ ^]^ investigated the effects of degradation for the ORR on Pt‐bimetallic catalysts, demonstrating that the dissolution of the less noble metal strongly affects catalyst stability, leaving an open question of whether similar effects play out in full membrane electrode assembly (MEA), where catalyst degradation evolves following different pathways. Additionally, for complementing analysis carried out at the nanoscale, scanning electron microscopy (SEM) of both MEA cross‐section and surface has been used to provide information about the heterogeneity of degradation, complementing the findings from SAXS.^[^ ^26^, ^27^ ^]^

As a tool to compare and quantify the stability of the different parts and materials composing the MEA, the US Department of Energy issued several accelerated stress test (AST) protocols.^[^ ^28^ ^]^ Among them, an AST was designed to alter the cathode electrocatalyst oxidation state to trigger catalyst‐related degradation mechanisms, such as dissolution and re‐deposition, agglomeration, and Ostwald ripening.^[^ ^21^, ^22^, ^29^ ^]^ Associated with these phenomena, it was found that the transport of metal ions is heavily influenced by the relative humidity (RH) and humidity gradients between the anode and cathode. While electrocatalyst degradation is well understood for the purely Pt catalyst, the durability and impact of the leached metal in Pt_3_Co remain unclear.^[^ ^27^, ^30^ ^]^ Specifically, its humidity dependency and spatial distribution along the full MEA could differ significantly from that in pure Pt catalysts.

This study adopts a multifaceted approach to delve into the complexities of humidity‐induced degradation in Pt_3_Co ORR catalysts by combining *operando* electrochemical characterization with *e*x situ techniques, notably SAXS and SEM, to chart the degradation effects meticulously. This methodology allows for an in‐depth exploration of the electrochemical behavior and facilitates the examination of morphological alterations in Pt_3_Co catalysts. Furthermore, it provides the means to compare these changes with those in Pt catalysts under different humidity conditions and spatial locations within the fuel cell, shedding light on the alloying influence.

## 2 Results and Discussion

The impact of relative humidity on Pt and Pt_3_Co catalysts, without acid treatment, is compared by employing a potential cycling accelerated stress test based on the cathode catalyst degradation test run at distinct operating conditions, in agreement with standards issued by the US Department of Energy,^[^ ^28^ ^]^ summarized in **Table** **1**. The decision to forego acid treatment for the Pt_3_Co catalyst is deliberate to investigate the direct impact of relative humidity on the ORR catalyst degradation and its implication on performance. Previous studies have shown that acid‐treated Pt‐alloy catalysts exhibit minimal leaching rates (≈3.4 µg_Co_ cm^−2^),^[^ ^31^, ^32^ ^]^ which can obscure the assessment of how leaching from the less noble metal (Co) impacts the durability and performance of PEFCs. AST1 and AST2 served each other to simulate different humidity conditions that are essential to evaluate the performance and durability of PEFC. AST1 imposes common relative humidity conditions during testing, allowing for the assessment of Pt and Pt_3_Co catalyst performance degradation in a controlled environment. In contrast, AST2 introduced a humidity gradient, mimicking the more realistic conditions encountered during PEFC operation. The differential response of Pt and Pt_3_Co catalysts in these two test scenarios are compared in order to understand the impact of relative humidity.

**Table 1:** AST operating conditions for both Pt and Pt_3_Co catalysts.

	Cell temperature [°C]	Anode dew point [°C]	Cathode dew point [°C]	Anode media	Anode min flow rate [nlpm]	Anode pressure [Atm]	Cathode media	Cathode min flow rate [nlpm]	Cathode pressure [Atm]
AST 1	80	80	80	H_2_	0.1	1	N_2_	0.3	1
AST 2	80	52.3	82.4	H_2_	0.1	1	N_2_	0.3	1

**Figure 1:**
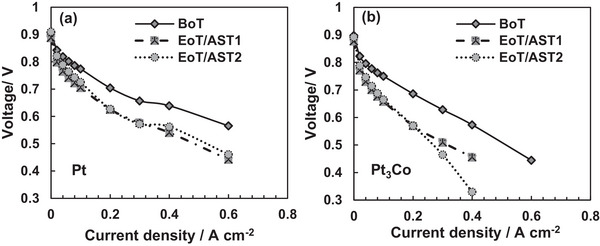
Polarization curves collected at the BoT and EoT for the two catalysts during the AST. Comparative analysis a) Pt catalyst and b) Pt_3_Co.

As a starting point for the analysis, polarization curves recorded before and at the end of stress are compared in **Figure** **1**, to show the influence of relative humidity on the Pt and Pt_3_Co catalysts degradation rates. When Pt catalyst is aged according to AST1 at 0.4 A cm^−2^ (Figure 1a), a voltage loss of 100 mV (equivalent to 16% loss) is observed at the end of the test (EoT) in comparison to the begin of test (BoT), highlighting performance degradation over time. Conversely, once AST2 are applied (characterized by a humidity gradient) to the Pt catalyst, the voltage loss measures 70 mV (representing 12% loss) at EoT. Despite being substantial, this loss is comparatively lower than that observed in AST1 under controlled humidity conditions when a Pt catalyst is used. With a Pt_3_Co catalyst aged at the same conditions (Figure 1b), a different response is evident: after AST1, a voltage reduction of 130 mV (representing 23% loss) is observed, indicating that Pt_3_Co catalysts are subjected to performance degradation in controlled humidity conditions, somewhat greater than Pt catalysts in similar conditions. In contrast, when a humidity gradient is present (AST2), a more substantial voltage loss is detected (230 mV, equivalent to 41% loss), emphasizing that humidity can significantly impact the performance of Pt_3_Co catalysts, resulting in more pronounced voltage losses. This underperformance of the Pt_3_Co is attributed to the amount of leached Co according to observation done by Greszler et al.,^[^ ^33^ ^]^ who noted a correlation between loss in catalyst mass activity and Co leaching within the catalyst layer. Several studies have further explained that after leaching, Co^2+^ ions exchange with proton in SO^₃−^H⁺ of the electrode ionomer, which increases kinetic losses, as well as proton and mass transport losses.^[^ ^4^, ^30^, ^34^, ^35^, ^36^ ^]^ These observations suggest that relative humidity gradient affects potential losses differently for Pt catalysts and Pt_3_Co catalysts. As a preliminary outlook, it can be underlined as Pt catalysts are less sensitive to humidity imbalances, resulting in lower voltage losses than Pt_3_Co.

**Figure 2:**
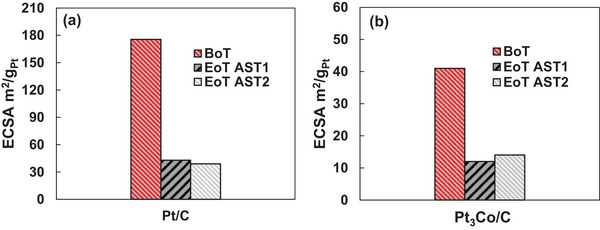
ECSA measured via integration of the H_upd_ area at the BoT and EoT for the two catalysts during the AST. Comparative analysis a) Pt catalyst and b) Pt_3_Co.

To assess changes in ECSAs, CV was measured at BoT and at EoT. Although the assumption of the H‐Pt monolayer may not be directly applicable to Pt_3_Co catalysts due to the presence of Co, the CV was used to compare changes in surface area due to degradation. As displayed in Figure S1 (Supporting Information), the areas of desorption hydrogen desorption decrease during PEFC operation for both the Pt (**Figure** **2**a) and Pt_3_Co catalyst (Figure 2b), indicating the loss of catalyst active sites. ECSAs of all MEAs decreased as calculated from the hydrogen desorption peak in the CV obtained with exposure to N_2_ at the cathode. During AST1 and AST2, the Pt catalyst exhibited ECSA losses of 75% and 78%, respectively, and for Pt_3_Co, ECSA was found to be reduced by 71% and 66%, respectively. ECSA reduction here reported was already addressed to be primarily due to the growth of catalyst nanoparticle size,^[^ ^37^ ^]^ supported in this work by the SAXS data, which can be induced by several phenomena within the operating regime, such as catalyst dealloying, dissolution, oxidation, redisposition, Ostwald ripening,^[^ ^6^, ^19^, ^38^ ^]^ as in these conditions, carbon support corrosion is expected to be less severe.

**Figure 3:**
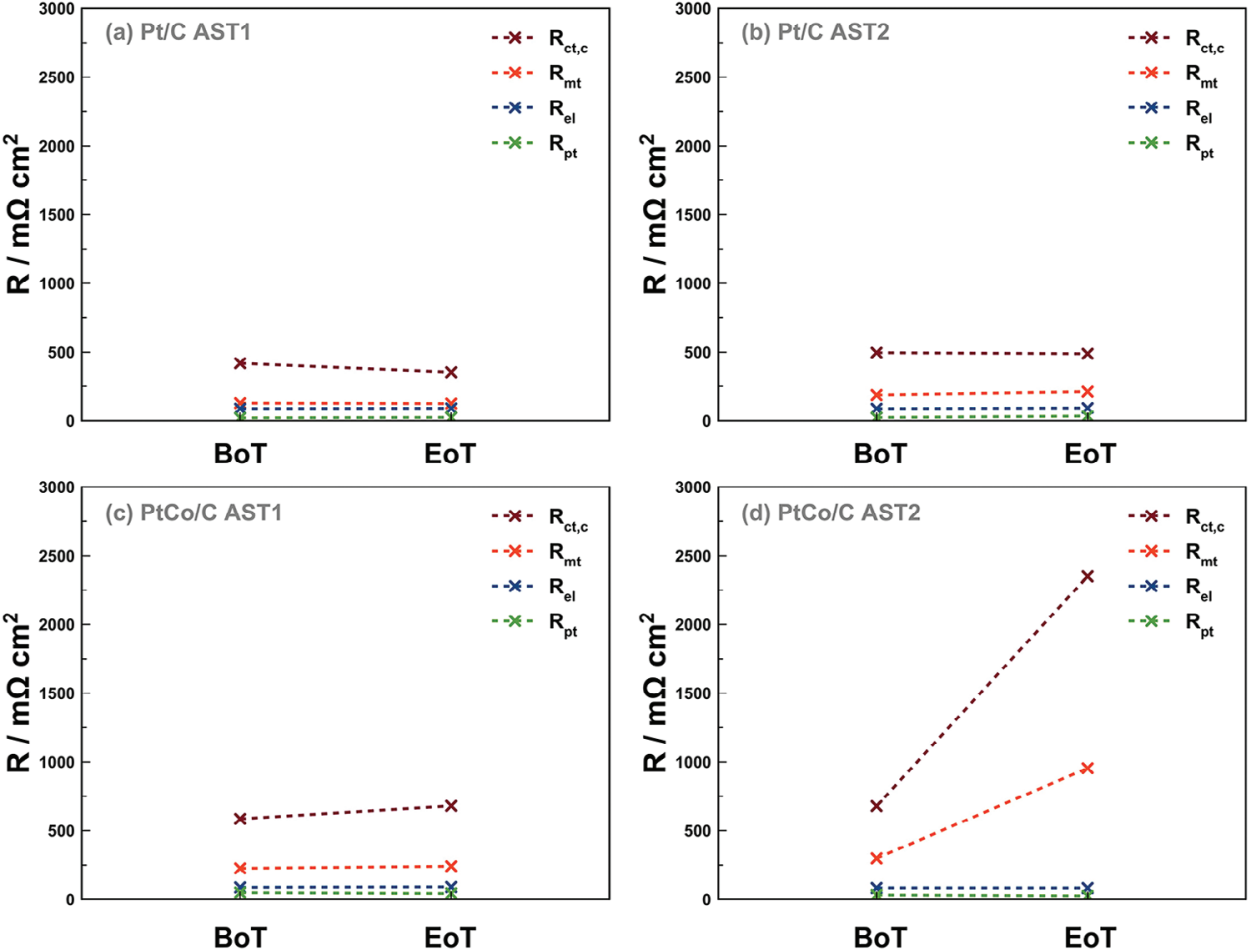
Resistance contributions calculated from the recorded impedance spectra. Change in resistances from BoT and EoT for Pt‐based MEAs with a) AST1 and b) AST 2. Change in resistances from BoT and EoT for Pt_3_Co‐based MEAs with c) AST1 and d) AST 2.

To further assess how changes in ECSA reflect on loss of efficiency of reaction kinetics, the variation of the single contributions to the total impedance of the MEA were retrieved by least square fitting EIS measurements (Figure 3; Figure S2, Supporting Information) by means of the equivalent circuit model (EC) presented in our previous works.^[^ ^4^, ^39^ ^]^ Here, the overall resistance (Table S1, Supporting Information) is composed by four resistances, configured either as bare resistances or in parallel to a capacitive term: the cathodic charge transfer resistance (*R*_ct,c_), the mass transport resistance (*R*_mt_), the membrane electrolyte resistance (*R*_el_), and the resistance of proton transport in the catalyst layer (*R*_pt_). For the Pt‐based MEAs, there were only minor changes during both ASTs. *R*_el_ and *R*_pt_ remained relatively stable, while *R*_ct,c_ decreased from 418.75 to 353.32 mΩ cm^2^ during AST1, and *R*_mt_ showed a small variation from 130 to 126.75 mΩ cm^2^. This unexpected result suggests that other factors, such as low intrinsic catalyst activity and suboptimal surface coverage, may contribute to the performance loss observed in the kinetic current region. During AST2, *R*_ct,c_ showed a minor decrease from 418.75 to 353.325 mΩ cm^2^, while *R*_mt_ increased from 187 to 212 mΩ cm^2^. As expected, a more pronounced variation was found when analyzing the cathode Pt_3_Co‐based MEAs. Here, during AST1, only *R*_ct,c_ increased from 585 to 680 mΩ cm^2^, while with AST2, both *R*_ct,c_, and *R*_mt_ increased dramatically from 678 to 2350 mΩ cm^2^ and 298 and 953 mΩ cm^2^ respectively. These results concur with polarization curve measurements since at 0.4 A cm^−2^ (operating point for EIS recording), the voltage loss is overall higher for Pt_3_Co‐based MEAs than the Pt‐based ones. Furthermore, when comparing voltage losses at EoT for both protocols applied to the Pt_3_Co‐based MEAs, it is visible that AST2 induced harsher degradation, in agreement with the previously reported voltage loss. From the form of the polarization curves, it is also visible that at EoT for AST2, the Pt_3_Co‐based MEA shows signs of nonlinear voltage drop at 0.4 A cm^−2^. Since the high‐frequency resistance remains relatively unchanged (see Figure S3, Supporting Information), the membrane's conductivity is likely unaffected. The issue may lie in the proton conductivity in the catalyst layer. Borup et al.^[^ ^40^ ^]^ demonstrated that the amount of Co^2^⁺ exchange influences proton conductivity and water uptake. Higher Co^2^⁺ exchange reduces water uptake in the catalyst layer ionomer, leading to more complex oxygen pathways and increased mass transport resistance.

**Figure 4:**
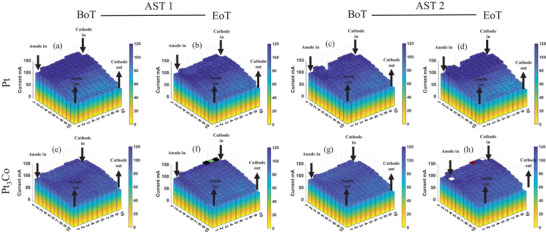
Evolution of locally resolved current distribution. Comparison of current distribution mapping recorded at 0.4 A cm‐2 at BoT and EoT for each AST. Data recorded from Pt‐based MEAs are represented on the first line: a) AST1/BoT, b) AST1/EoT, c) AST2/BoT, d) AST2/EoT. Data recorded from the Pt_3_Co‐based MEAs are represented on the second line: e) AST1/BoT, f) AST1/EoT, g) AST2/BoT, and h) AST2/EoT.

Being known that reactant distribution over the surface of the MEA is hardly homogenous, and due to the fact that selected ASTs are characterized by different water distribution profiles across the MEA, we wanted to investigate differences in degradation according to the position of the catalyst layer with respect the inlets and outlets of reactants. For this purpose, the local current distribution at a polarization current of 0.4 A cm^−2^ was analyzed to assess the heterogeneity of degradation across the surface area of the MEA. In **Figure** **4**, the local current distribution in Pt‐based (Figure 4a–d) and Pt_3_Co‐based MEAs (Figure 4e–h) are illustrated. The Pt‐based MEAs show a consistent current distribution pattern in both ASTs, providing a reliable baseline for comparative analysis. In contrast, Pt_3_Co‐based MEAs exhibit a notable difference already at BoT, where a shift in current toward the cell center is observable. This behavior is suggested to be due to catalyst dealloying and Co leaching: in fact, dealloying induces localized changes in the composition of the catalyst material, thereby altering its electrochemical activity^[^ ^10^, ^41^ ^]^ and resulting in uneven current distribution across the catalyst surface. Concurrently, Co leaching, as result of dealloying, generates defects or voids at the nanoscale,^[^ ^42^, ^43^ ^]^ disrupting electrochemical pathways and further contributing to non‐uniform current distribution. When comparing the results from AST1 and AST2, the effects of humidity on Pt_3_Co‐based MEAs become apparent. The potential influence of Co leaching emerges as a pivotal factor, raising questions about the intricate interplay of dealloying and the current distribution. Consequently, the pronounced potential loss and mass transfer resistance observed for the Pt_3_Co‐based MEAs in this context gains further validation, emphasizing the nuanced relationship between alloy composition, changes in ECSA, and the resulting constraints on oxygen supply to specific regions of the Pt catalyst. These factors lead to spatial variations in electrochemical activity, conductivity, and oxygen accessibility across the catalyst surface, ultimately influencing the observed current distribution at larger scales.

**Figure 5:**
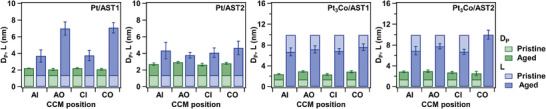
Post‐mortem variation of catalyst morphology. For the four aged samples, mean particle diameter (D_P_) and structure factor cut‐off distance (L) are compared concerning their values in pristine conditions (dark and light colors, respectively).

To better observe the effects of the different current distributions on catalyst morphology, SAXS patterns recorded from MEAs in pristine conditions were compared with SAXS post‐mortem analysis of the four MEAs. As detailed in the Experimental Section, a previously developed analytical model^[^ ^44^ ^]^ was used to fit the recorded scattering patterns to retrieve information about catalyst particle size distribution and their degree of aggregation. Particle size distribution was described in terms of mean particle diameter, D_P_, and width, σ_P_, within the Schulz distribution.^[^ ^45^, ^46^ ^]^ As also done by Pavia and co‐workers,^[^ ^47^ ^]^ aggregation was described in terms of the average cut‐off distance, L (which is a qualitative marker, dependent on the extension of the aggregate), and fractal dimension, D_f_ (describing the degree of aggregation). In pristine conditions (Tables S2 and S3, Supporting Information), the mean particle size of Pt catalyst and Pt_3_Co catalysts was found to be equal to 1.38 ± 0.04 and 1.73 ± 0.02 nm, respectively, while the standard deviation was found to be similar for the two catalysts. From the analysis of the degree of aggregation, Pt catalyst nanoparticles showed to form smaller clusters with respect to Pt_3_Co ones, as the cut‐off distance was found to equal to 2.05 ± 0.22 nm for Pt catalyst and to 9.94 ± 0.53 nm for the Pt_3_Co one. In both cases, a fractal number close to or slightly larger than two was retrieved, indicating that particles are arranged in mass fractals, which are representative of compact clusters.^[^ ^48^ ^]^ The difference in aggregation size was found in agreement with the scattering patterns, as indicated by the different displacements of the shoulder originating from the structure factor of the two samples measured in pristine conditions (and highlighted by the arrows in Figure S4, Supporting Information). Aged CCMs were scanned near gas inlets and outlets where the most visible effects of AST were expected to be found, as detailed in the Experimental Section. The main results are shown in **Figure** **5**, while a quantitative comparison is summarized in Tables S1 and S2 (Supporting Information). As a main outlook, a part from of an overall increase of both particle size and cut‐off distance, no clear trend could be identified in the function of the protocol used or catalyst composition. Concerning Pt nanoparticles, after running AST1 an average doubling of particle size was revealed, with no remarkable variations of particle standard deviation. Conversely, while such increase in particle size was slightly enhanced after AST2, an overall reduction of particle standard deviation was detected, which was more pronounced in the proximity of the inlets than the outlets. The reported difference in growth could be related to the different environments in which degradation occurred. From the structure factor analysis, in all of the cases, the fractal dimension ranges between three and four, underlying as the nature of the aggregate changes from mass fractal to surface fractal,^[^ ^48^, ^49^, ^50^, ^51^ ^]^ allowing to suppose that particles are forming compact aggregates characterized by a rough surface clarifying the loss of ECSA as demonstrated by Bogar et al in their previous works.^[^ ^44^, ^52^ ^]^ Nonetheless, the most remarkable variations involve the cut‐off distance. Concerning AST1, cut‐off distance evolution was found limited in proximity of the gas inlets (where an increases of ≈1.6 nm was reported), and more pronounced in proximity of the outlets (reaching values ≈7 nm), in agreement with the higher mobility expectable in a more water‐rich environment. Conversely, AST2 induced more pronounced particle growth and a reduced cut‐off distance. Such trends were further confirmed by fitting the scattering patterns retrieved from the vertical scan of the CCMs in their middle position, where the average value of the four main parameters over the Region Of Interest (ROI) in proximity of gas inlets and outlets (Tables S1 and S2, Supporting Information) match with the fit results retrieved from fitting the scattering patterns along the middle scan (Figure S5, Supporting Information). When focusing on Pt_3_Co catalyst nanoparticles, a homogenous growth in mean particle size and standard deviation was revealed with no remarkable variation among gas inlets and outlets as well as in the function of RH. In analogy with bare Pt catalysts, the nature of the fractal aggregate mutated toward a more compact aggregate showing a rough surface. After applying AST1, the average cut‐off distance was diminished from ≈10 to ≈7 nm, and an analogous trend was reported also after running AST2. In this extent, Co leaching from the sample could be one of the main phenomena leading to aggregate fragmentation or shrinking, as opposed to bare‐Pt‐based samples.

**Figure 6:**
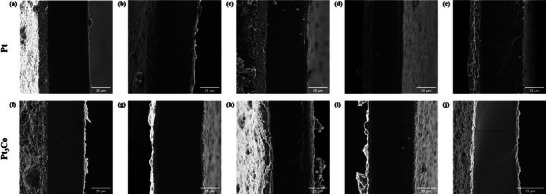
Cross‐section SEM images of the cathode(left), PEM, and anode(right) of pristine and degraded CCMs. In the first line, CCMs loaded with Pt catalyst are shown. a) pristine condition; aged by running AST1: b) middle top section, c) middle bottom section; aged by running AST2: d) middle top section, e) middle bottom. On the second line, CCMs loaded with Pt_3_Co catalyst are shown. f) pristine conditions, aged by running AST1: g) middle top section; h) middle bottom section; aged by running AST2: i) middle top section, and j) middle bottom section.

**Figure 7:**
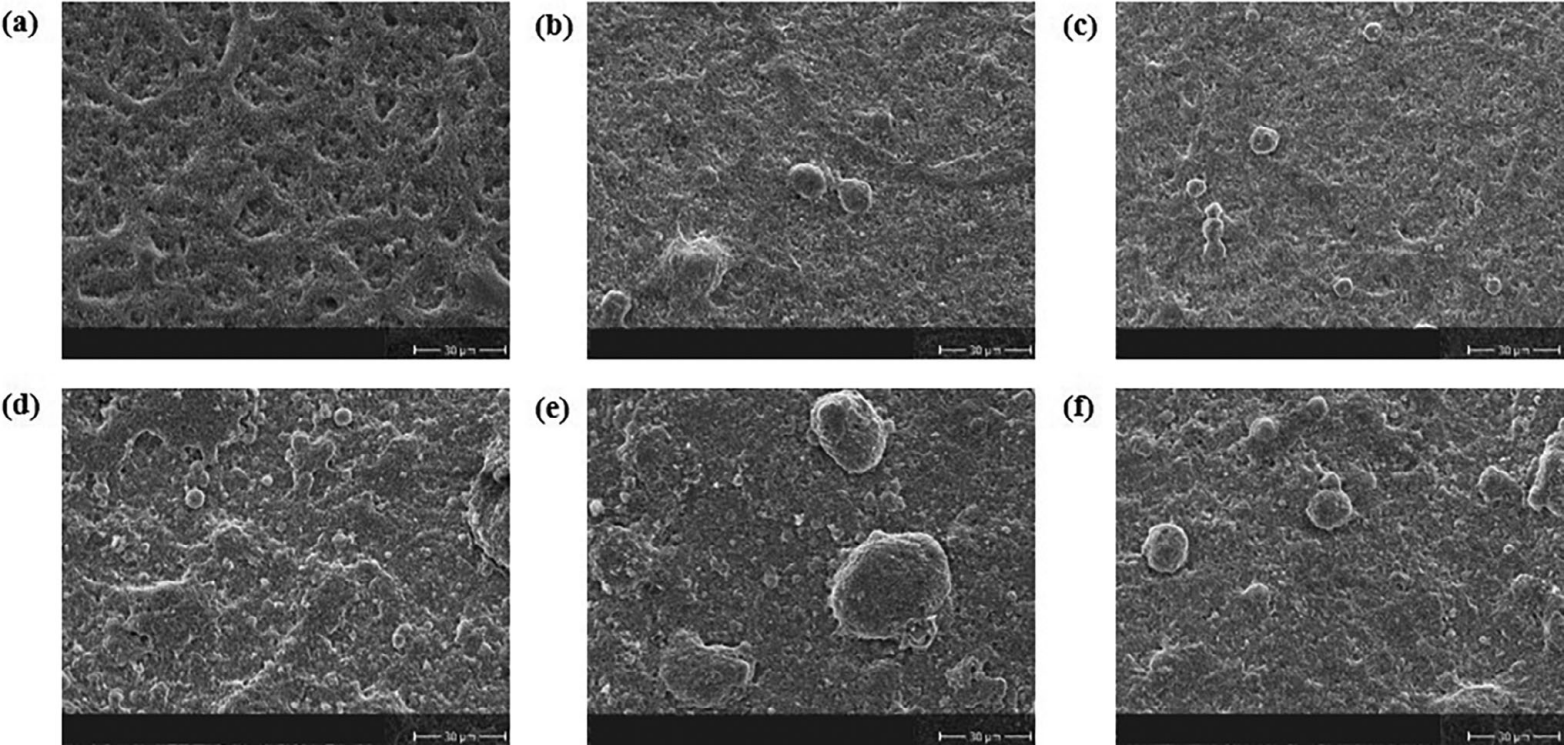
SEM images of Pristine and degraded CCMs surface. a) pristine Pt catalyst; b) AST1 middle top section Pt catalyst; c) AST2 middle top section Pt catalyst; d) pristine Pt_3_Co catalyst; e) AST1 middle top section Pt_3_Co catalyst; f) AST2 middle top section Pt_3_Co catalyst.

**Figure 8:**
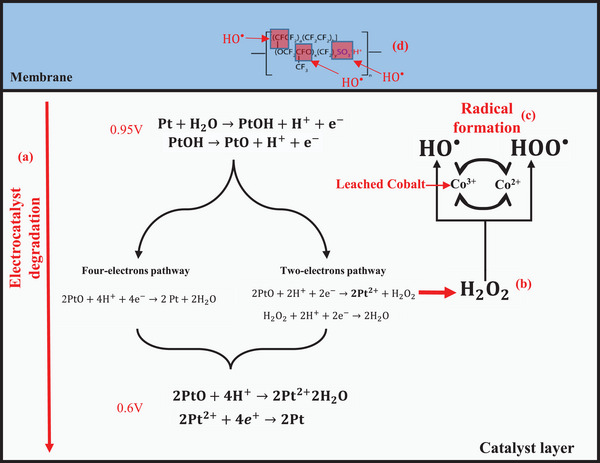
Schematic illustrations of the suggested mechanism underlying membrane chemical degradation using a Pt_3_Co catalyst. a) Pt electrocatalyst degradation; b) H_2_O_2_ formation; c) radical formation, and d) membrane chemical oxidation.

Finally, with the aim of linking morphological and electrochemical analysis, SEM was utilized to analyze the macroscopical morphological alterations induced by ASTs. Here, pristine and aged CCMs were observed through cross‐sectional and surface scans, as depicted in **Figures** **6**a–j and **7**a–f, respectively. SEM images showed the cathode catalyst layers of Pt‐based MEAs (Figure 6a–e) and Pt_3_Co‐based ones (Figure 6f–j). Analyses of cathode catalyst layer and membrane thicknesses are summarized in **Table** **2**. Catalyst layer thicknesses calculation showed insignificant thinning for Pt‐based MEAs, with thickness ranging from 12.62 to 12.02 µm. Conversely, Pt_3_Co‐based MEAs exhibited thinning, ranging from 4.81 to 4.2 and 3.08 µm after having applied AST1 and AST2, respectively. This degradation was attributed to Co leaching at high humidity conditions, leading to increased mobility of the leached Co metal. Initially, the leached Co may exists in the form of ions, specially Co^2+^ and Co^3+^.^[^ ^31^, ^40^ ^]^ These can undergo reduction reactions to form metallic Co particles near the electrode surfaces. Several factors such as applied voltage, hydrogen crossover, and high humidity can promote this phenomenon.^[^ ^53^, ^54^ ^]^ Once formed, Co particles can demonstrate mobility within the MEA and potentially wash out of the system as evidence of 0.28 µg_Co_L^−1^ (Table S4, Supporting Information) of Co were detected via ICP‐MS when analyzing the cathode effluent water collected once running AST2 on Pt_3_Co‐based MEA. This finding elucidates the observed decrease in catalyst layer thickness and confirms the occurrence of Co leaching. Additionally, membrane thickness was examined for all MEAs, with Pt‐based MEAs showing homogeneity and no thinning, consistent with prior studies.^[^ ^21^, ^55^ ^]^ In contrast, Pt_3_Co‐based MEAs exhibited heterogeneity in membrane thickness, indicating localized chemical degradation that may be caused by reactions between leached cobalt and byproducts such as H_2_O_2_ in the cathode. During the catalyst degradation process as suggested by Khedekar et al.,^[^ ^21^ ^]^ a pristine Pt surface is present at the lower potential limit. In the presence of water, as the potential is increased, Pt dissolution occurs at defect sites at a low rate. As the potential crosses 0.8 V, OH groups adsorb on the Pt surface, ultimately leading to PtO formation. Subsequently, PtO is reduced as it reacts with protons to form dissolved Pt and water, as illustrated in **Figure** **8**. During this last step, the ORR may proceed through either a highly efficient four‐electron pathway or a sluggish two‐electron pathway, resulting in H_2_O_2_ production. The generated H_2_O_2_ may then be converted to oxidative radicals through a Fenton‐like reaction upon contact with Co ions,^[^ ^56^, ^57^ ^]^ triggering membrane chemical degradation and clarifying the observed thinning. Surface observations further confirmed structural changes, with all MEAs exhibiting distinct surface roughness and evident growth in certain areas compared to pristine, as displayed in Figure 7.

**Table 2:** Catalyst layer and membrane thicknesses of all MEAs acquired with the cross‐sectional SEM for the pristine and aged samples.

		Pristine	AST1	AST2
Catalyst layer thickness µm	Pt	12.62 ± 0.38	12.02 ± 0.88	12.13 ± 0.93
	Pt_3_Co	4.81 ± 0.51	4.20 ± 0.60	3.08 ± 0.61
Membrane thickness µm	Pt	49.72 ± 0.53	48.40 ±1	51.24 ± 0.82
	Pt_3_Co	49.24 ± 0.96	46.86 ± 1.28	44.96 ± 1.55

## 3 Conclusion

This study investigates the influence of relative humidity gradients on the performance and degradation mechanisms in PEFCs under ASTs at different humidification conditions comparing their impact on Pt and Pt_3_Co catalysts. By comparing the polarization curves, we could report significant voltage losses after running both AST1 and AST2.In particular, Pt_3_Co catalysts was interested in more pronounced losses of performance (particularly after AST2). Cyclic voltammetry analysis reveals substantial losses in ECSAs for both catalyst types. Electrochemical impedance spectroscopy analysis illustrates significant changes in resistance contributions, especially for Pt_3_Co‐based MEAs, suggesting mass transfer limitations and increased charge transfer resistance. Local current distribution analysis revealed distinctive behaviors between Pt and Pt_3_Co catalysts, with Pt_3_Co‐based MEAs exhibiting activation impacts attributed to initial leaching phenomena. Of particular significance, post‐mortem SAXS analysis confirms a significant increase in particle sizes for both Pt and Pt_3_Co catalysts. Notably, degraded Pt_3_Co‐catalyzed MEAs exhibit reduced cluster sizes compared to the pristine state, supporting the observed structural degradation. This SAXS data provides crucial evidence of the morphological changes occurring within the catalyst materials under the influence of relative humidity gradients during ASTs. SEM analysis of cathode catalyst layers and membrane thickness indicates substantial thinning and heterogeneity in Pt_3_Co‐based MEAs, attributed to cobalt leaching and membrane degradation, especially after AST2. Although not substantial, this represents the initial observation of membrane thinning under these conditions, prompting the need for further exploration. The impact of relative humidity on catalyst performance and degradation mechanisms is elucidated, and the results clearly show that increased water content in the cathode catalyst layer accelerates cobalt dissolution from Pt_3_Co catalysts. Under humidity gradients, uneven water distribution influences cobalt dealloying, platinum dissolution, and potentially Ostwald ripening rates. The latter, stands out as the predominant mechanism driving degradation under this specific condition. By gaining this understanding of the mechanisms driving degradation, future research can focus on developing strategies to mitigate Ostwald ripening and improve the long‐term performance of Pt‐alloy catalysts.

## 4 Experimental Section

### Catalyst Materials and Catalyst‐Coated Membrane (CCM) Fabrication

Two different catalysts were used to produce electrocatalyst inks. These catalysts consist of Pt catalyst and Pt_3_Co materials with metal loadings of 20 and 30 wt.% obtained from Thermo Scientific (Kandel) GmbH (Germany) and Sigma‐Aldrich, ACS Reagent (USA) respectively. It was worth noting that the Pt_3_Co catalysts used in this study were intentionally not pre‐leached before the ink‐making step. This decision was made to investigate the leaching dynamics during ASTs. To ensure consistent proton transport and water management, the ionomer content in the ink (5 wt.% solution solutions, NS‐5 QuinTech) was kept constant at ≈30 wt.% of the dry electrocatalyst mass. This achieved an I/C ratio of 0.6 for Pt/C, while the Pt_3_Co/C reached an I/C ratio of 0.9. A stable ink formulation required mixing 2‐propanol (Honeywell, Chromasolv for HPLC, ≥99.9%) and water (Milli‐Q) at a ratio of 0.97:0.03 for Pure‐Pt, adjusted to 0.85: 0.15 for Pt_3_Co/C. All catalyst inks were sonicated for 45 min before catalyst‐coated membrane (CCM) fabrication. All CCMs were ultrasonic spray‐coated using an ExactaCoat OP3 ultrasonic spray coater (Sono‐Tek Corporation, Milton, NY, USA), which had a 120 kHz nozzle that controls precise thickness. A NM212 Nafion membrane sourced from QuinTech with a thickness of 50.8 µm was chosen for CCM preparation, attaching the membrane to a porous PTFE filter by vacuum suction, and heating to 100 °C. The catalyst ink was applied in a serpentine pattern to achieve loadings of 0.03 mg_Pt_cm⁻^2^ on the anode and 0.125 mg_catalyst_cm⁻^2^ on the cathode. The anode uses a Pt catalyst, while the cathode could use either Pt or Pt_3_Co. The 0.03 mg_Pt_cm⁻^2^ loading on the anode was specifically chosen to facilitate SAXS analysis of the cathode catalyst, allowing for better characterization of the ORR catalyst.

### Fuel Cell Construction and Experimental Setup

The MEAs were constructed using the prepared CCMs alongside a commercially available gas diffusion layer (GDL), specifically Sigracet 22 BB carbon‐coated carbon paper. Stress test conditions are reported in Table 1. Current distributions were measured using a segmented current measurement sensor plate (Current Scan, S++, Germany) inserted between the cathode bipolar plate and the collecting plate. The series of shunt resistors made it possible to measure the current over a 10  ×  10 array. The signals acquired were directed to a multiplexer and the A/D converter. The hardware configuration comprises a 25 cm^2^ single cell with triple serpentine flow fields on both anode and cathode in a semi‐co‐flow configuration. All electrochemical experiments were conducted using an automatically controlled fuel cell station (G60, Greenlight Innovation, Canada), and an electrochemical workstation (Reference 5000, Gamry, USA) was employed for CV and EIS measurements.

### Electrochemical Measurements and Diagnostics

Before conducting ASTs, the fuel cells underwent activation according to the Joint Research Centre (JRC) protocol.^[^ ^58^ ^]^ Following activation, polarization curves, EIS, and CV measurements were conducted. This analytical framework provided a detailed assessment of performance degradation throughout the operational lifespan of the cells. For polarization testing, the established JRC protocol was modified. Both anode and cathode gases were humidified to attain 100% relative humidity at an operational temperature of 80 °C. Gas pressures were maintained at 50 and 30 kPa for the anode and cathode, respectively, with stoichiometric ratios set at 1.5 for the anode and 3 for the cathode. Electrochemical impedance spectroscopy (EIS) measurements were performed in the frequency range of 0.3 Hz–30 kHz, with an Amplitude of 5% (10% at an operating point of 0.4 A cm^−2^) using H_2_/air, λ = 1.5/3, p = 50/30 kPa(g), 100%RH and T_cell_ = 80 °C. From the recorded impedance spectra, the resistance contributions to the overall cell resistance were calculated by the same equivalent circuit (EC) modeling approach as described in detail in a previous publication with the same EC model.^[^ ^4^, ^39^ ^]^ In short, first, the distribution of relaxation time was calculated with the open source MATLAB GUI “DRTtools,” programmed by T.H Wan, F. Ciucci et al.^[^ ^59^, ^60^ ^]^ This was used to determine the characteristic frequencies of each electrochemical process: Cathode charge transfer, catalyst layer proton transport, and mass transport. The corresponding plots showing the peaks are presented in Figure S1 (Supporting Information). The initial model parameters for the fitting algorithm were calculated using the frequency value and the estimate of real axis values defined by the intercepts of the semi‐circles drawn in the Nyquist plot with the maximum at the corresponding characteristic frequency. The fitting was performed using the Zview software (Scribner). For the CV, hydrogen and nitrogen gases were supplied to the anode and cathode, respectively, while maintaining 100% humidity and an operating temperature of 80 °C. The flow rates of H2 and N2 were maintained at 0.1/0.3 nlpm, respectively. CV measurements featured a sweep rate of 50 mV/s.

As the U.S. Department of Energy recommended, a durability protocol tailored to its properties was adopted to assess the cathode catalyst's resilience. The catalyst above ASTs, AST 1 and AST 2, were executed under specified operating conditions outlined in Table 1. These protocols involved subjecting the cells to 30 000 cycles of square‐wave voltage oscillations spanning from 0.6 to 0.95 V. Each voltage phase featured a 2.5°s dwell time, accompanied by a 0.5°s ramp time, resulting in a complete cycle duration of 6°s.^[^ ^28^ ^]^ These durability assessments were performed using 25 cm^2^ membrane electrode assemblies (MEAs) operating under H_2_/N_2_ flow conditions at 80 °C and ambient pressure, with meticulous control of relative humidities.

### Small Angle X‐Ray Scattering (SAXS)

Small‐angle X‐ray scattering (SAXS) patterns were collected on the Austrian SAXS beamline at the ELETTRA synchrotron in Trieste, Italy.^[^ ^61^ ^]^ After removing GDLs from MEAs, CCMs were measured ex situ in transmission mode at room temperature and pressure. Silver behenate was used as a calibrant. Sample‐to‐detector distance was set to 2001.87 mm, while the beam energy was set to 16 keV, resulting in a q‐range extending from 0.087 to 7.715 nm^−1^. A 2D‐pixel detector (Pilatus3 1 M, Dectris) was used to collect 2D scattering patterns, which were radially integrated (SAXSDOG software^[^ ^62^ ^]^). Data crunching and data analysis were performed using IGOR Pro software (IGOR Pro 7.0.8.1, Wavemetrics). The beam size was set to 0.5 mm x 0.5 mm, to guarantee enhanced spatial resolution. The single layers composing the CCMs (Nafion and the bilayer composed by Nafion and Vulcan support) and the CCMs in pristine conditions were characterized by recording 200 scattering patterns distributed over a scanning area of 4 mm^2^, with a horizontal resolution of 1 mm and a vertical resolution of 0.2 mm. Conversely, aged CCMs were scanned in six different areas (with the same resolution), considered of interest with respect to the used AST. 500 scattering patterns were recorded while mapping a 1 cm^2^ area in proximity of the anode inlet (AI), anode outlet (AO), cathode inlet (CI), and cathode outlet (CO) of the fuel cell. In addition, a partial middle scan in the upper‐central part of the CCM and a complete middle (MS) scan along the vertical direction of the whole CCM were also performed, using the same spatial resolution. From the collected scattering patterns, scattering correlation length (ξ), defined as the mean width of the correlation function γ_0_(*r*) among the scattering bodies,^[^ ^63^, ^64^ ^]^ was calculated as the ratio among the first and the second moment of the scattering curve, integrated within a finite q‐range: $\xi =\pi \int_{q_{\min}}^{q_{\max}}I\left(q\right)\mathrm{qdq}/\int_{q_{\min}}^{q_{\max}}I\left(q\right)q^{2}\mathrm{dq}$, with *q*_*min*_ = 0.157 nm^−1^ and q_max_ = 2.027 nm^−1^. Due to the sensitivity of this quantity to structural changes,^[^ ^65^ ^]^ the scattering correlation length was used to highlight any size variations over the scanned areas of the aged CCM. Correlation‐length maps were built to this extent, as depicted in Figure S6 (Supporting Information). Quantitative SAXS analysis was subsequently carried out using least square fitting the scattering patterns obtained from the patterns of the pristine CCMs and the Regions Of Interest (ROI) of the CCMs considered to be most representative of each sample (framed by the yellow squares in Figure S6 (Supporting Information), as depicted in Figure S7, Supporting Information). The analytical model used for fitting was built by considering all of the layers composing the CCM, previously characterized, as already done on a similar system^[^ ^44^ ^]^: the Nafion membrane was represented by two Voigt peaks,^[^ ^66^, ^67^ ^]^ the Vulcan support was modeled by mean of a power law and the catalyst nanoparticles were represented as a set of nanoparticles following the Schultz distribution^[^ ^45^ ^]^ clustered together forming aggregates^[^ ^47^ ^]^: *I*(*q*)∝*I*_*P*_ · *P*(*q*, *D*_*P*_,*σ*_*P*_) · *S*(*q*, *L*, *D*_*f*_), where I_P_ is the value of the catalyst forwarded scattering probability, D_P_ and σ_P_ are respectively the mean particle size and width within the Schultz distribution, L is the cut‐off distance describing the behavior of the pair correlation function for distances bigger than the size of a catalyst nanoparticle (which could be related to the average cluster size), and D_f_ is the fractal dimension.^[^ ^48^, ^68^ ^]^ According to the type of fractal aggregate, the fractal dimension could describe a mass fractal (1 ≤  *D*_*f*_ = *D*_*m*_  < 3) or a surface fractal (for a 3D system: d = 3, and with 3 ≤ *D*_*f*_ < 4, it results: 2 ≤ *D*_*S*_ < 3).^[^ ^48^, ^50^, ^51^, ^64^ ^]^ While in the last decades, it was demonstrated the existence of the so‐called cross‐correlation term generated by the mutual interference among support and decorating catalyst nanoparticles, more recent studies highlighted that for electrodes for fuel cell systems, the impact of such a term could be neglected in some circumstances,^[^ ^44^, ^69^ ^]^ such as low particle loading. By referring to the works of Gommes and co‐workers^[^ ^69^, ^70^ ^]^ in Supporting Information (dedicated chapter and Figure S8, Supporting Information), the influence of the cross‐correlation term on the total forwarded scattering probability was estimated, and the error introduced if this term was neglected was estimated to be low enough not to disrupt the SAXS analysis. Due to the benefits of computational resources and ease of modeling, the incoherent scattering sum among support and catalyst nanoparticles was performed, allowing to use an analytical model as a form factor. With the aim of highlighting any morphological variation in the function of its position in the MEA, the scattering patterns of each data point within the ROI around the inlets and outlets of the fuel cell were analyzed. The main parameters from the analytical model for the four aged CCMs are plotted in Figures S9–S12 (Supporting Information). In order to connect the results to the electrochemical parameters recorded from the segmented cell, data displayed in Figures S9–S12 (Supporting Information) were averaged 1 and listed in Tables S1 and S2. In addition, the fit results from the complete scan along the vertical direction are displayed in Figure S5 (Supporting Information).

### Scanning Electron Microscopy (SEM) and Energy‐Dispersive X‐Ray (EDX) Spectroscopy

The preparation of fresh and aged samples for SEM analysis involved both surface morphology scanning and cross‐sectional examination. Specifically, sections measuring 0.5 cm × 0.5 cm were excised from the middle portion of the MEA, taken from both the top and bottom surfaces, to enable surface scanning. In parallel, samples of identical dimensions were extracted from these positions for cross‐sectional analysis. These cross‐sectional samples were cryogenically treated by immersion in liquid nitrogen, followed by cryo‐cutting with a blade to ensure precise sectioning. For SEM analysis, a scanning electron microscope (Philips FEI XL 20) equipped with an Energy‐dispersive X‐ray spectrometer (EDX; remX GmbH, Germany) was utilized. The imaging of both surface and cross‐sectional views using a standard secondary electron (SE) detector operating at 10 kV with a probe current of 20 µA was within typical operating parameters for SEM analysis. These conditions were chosen to optimize image quality and resolution while minimizing sample damage. Catalyst layer and membrane thickness measurements were carried out using ImageJ. Multiple samples for the pristine and locations across the entire length of the cross‐section were measured for all CCMs.

### Inductively Coupled Plasma Mass Spectrometry (ICP‐MS) Analysis

The concentrations of cobalt in the samples were determined using a PerkinElmer Elan DRCe ICP‐MS. This instrument utilizes dynamic reaction cell (DRC) technology to effectively minimize polyatomic interferences. Calibration was performed externally over a range of 0.1 to 5 µg L^−1^, with 69Ga used as an internal standard at 10 µg L^−1^. The ICP‐MS was optimized for a peak signal at 115 In, maintaining oxide and doubly charged ion ratios below 3%. Sample introduction was achieved using a standard torch with a crossflow nebulizer, a Scott‐type spray chamber, and nickel cones. The limit of quantification (LOQ) was 0.2 µg L^−1^. Three samples from each type were analyzed to assess consistency and repeatability. Results were reported in micrograms per liter ( µg L^−1^).

## Conflict of Interest

The authors declare no conflict of interest.

## Supporting information



Supporting Information

## Data Availability

The data that support the findings of this study are available in the supplementary material of this article.
