# Supplementary material for: Humidity‐Induced Degradation Mapping of Pt3Co ORR Catalyst for PEFC by In‐Operando Electrochemistry and Ex Situ SAXS
Source: Small. 2024 Oct 16;20(51):2407591. doi: 10.1002/smll.202407591 (PMC11656675; doi:10.1002/smll.202407591)
Supplement: Supplementary file 1 — Supporting Information [file SMLL-20-2407591-s001.docx]

Supporting Information

Humidity-Induced Degradation Mapping of Pt_3_Co ORR Catalyst for PEFC by In-Operando Electrochemistry and Ex-Situ SAXS.

Joel Mata Edjokola*, Marco Bogar, Maximillian Grandi, Rodolfo Taccani, Heinz Amenitsch, Marjan Marinsek, Viktor Hacker and Merit Bodner

**Supplementary Figures**


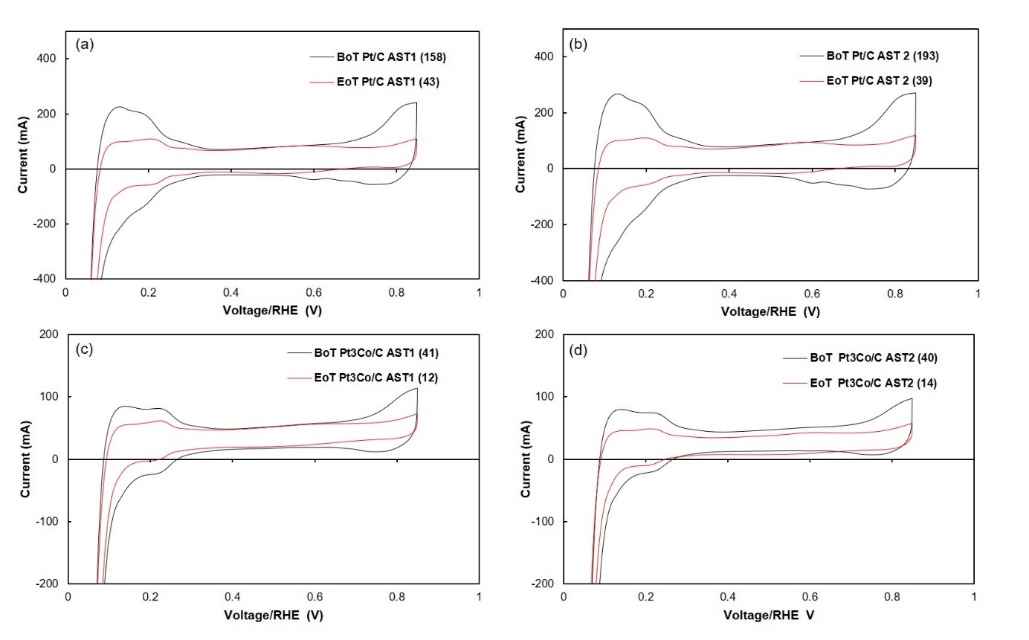


**Figure S1: CV characterization results for BoT and EoT.** (a) Pt-based AST 1; (b) Pt-based AST2; (c) Pt_3_Co-based AST1; and (d) Pt_3_Co-based AST2.


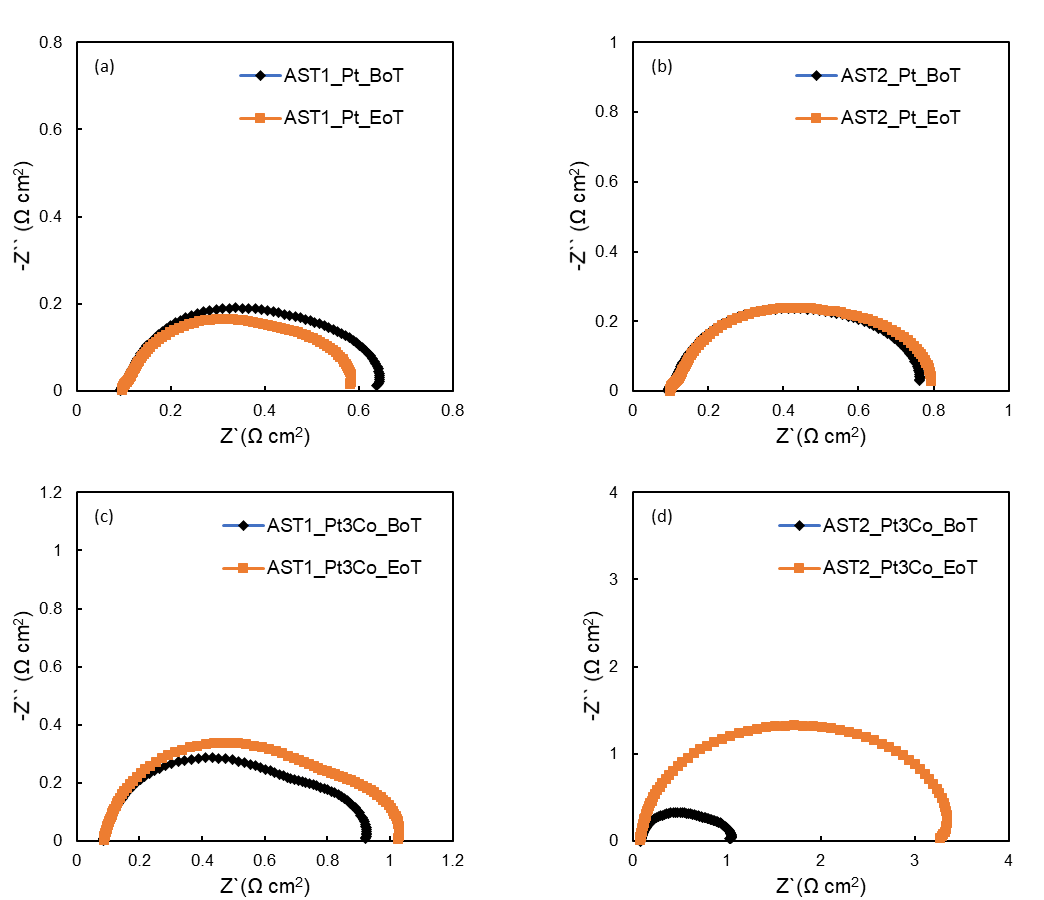


**Figure S2: EIS experimental data:** (a) Pt-based AST 1; (b) Pt-based AST2; (c) Pt_3_Co-based AST1; and (d) Pt_3_Co-based AST2.


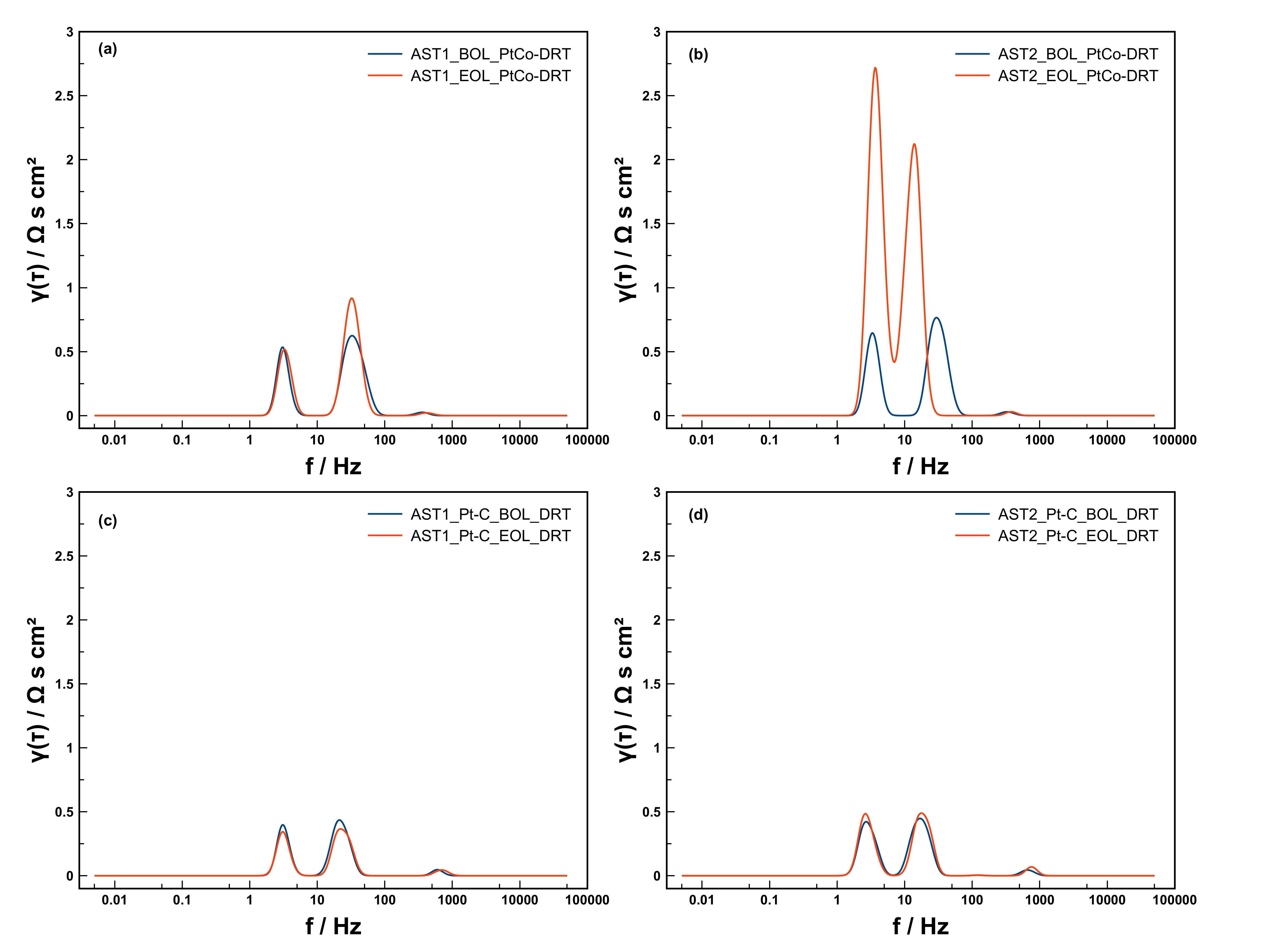


**Figure S3: DRT analysis results for BoT and EoT.** (a) Pt_3_Co-based AST1; (b) Pt_3_Co-based AST2; (c) Pt-based AST 1; and (d) Pt-based AST2.

**SAXS modeling: the cross-correlation term.**

By repeating the analysis previously carried out at a different catalyst loading^[44]^, the impact of the cross-correlation term was evaluated by following the framework provided by Gommes and co-workers^[69]^. The cross-correlation term can be neglected with low particle loadings if:

$$\frac{{\Delta\rho}_{P}\phi_{P}}{1-\phi_{S}}\ll{\Delta\rho}_{S}$$

Where ${\Delta\rho}_{S}$ and ${\Delta\rho}_{P}$ are the scattering contrast of the support and decorating particles respectively, while $\phi_{P}$ is the particle volume fraction. Such equation allows to define an upper limit in evaluating the weight of the cross-correlation term on the total forwarded scattering, which was quantified to be equal to about 13% with a catalyst load of bare Pt 20 wt% on a Vulcan XT72 substrate^[69]^. In this extent, to estimate the weight of the cross-correlation term the deconvoluted components modeling the support ($I_{S}\propto{\Delta\rho}_{S}^{2}\int{dr}_{S}r_{S}^{6}f_{S}\left( r_{S} \right)F^{2}\left( q,r_{S} \right)$), the catalyst population ($I_{PP}\propto N_{P}{\Delta\rho}_{P}^{2}\int{dr}_{P}r_{P}^{6}f_{P}\left( r_{P} \right)F^{2}\left( q,r_{P} \right)S\left( q,D_{f},L \right)$), the cross-correlation term ($I_{SP}\propto2N_{P}{\Delta\rho}_{P}{\Delta\rho}_{S}\int{dr}_{P}r_{P}^{3}f_{P}\left( r_{P} \right)F\left( q,r_{P} \right)\int{dr}_{S}r_{S}^{3}f_{S}\left( r_{S} \right)F\left( q,r_{S} \right)S_{P}\left( q,r_{S}+r_{P} \right)$), and their sum ($I_{tot}\propto I_{S}+I_{PP}+I_{SP}$) were compared for both catalyst formulations, and are represented in Figure S5 The subscripts *S* and *P* respectively refer to the substrate and the decorating catalysts nanoparticles, while${\Delta\rho}_{x}$ is the scattering contrast. $f_{x}\left( r_{x} \right)=\left( r_{x},R_{x},\sigma_{x} \right)$ is the probability distribution function within the log-normal distribution (with R_x_ the means particle radius and σ_x_ the distribution width), and N_P_ is the average number of decorating particles per support particle. $F\left( q,r_{x} \right)$ is the scattering amplitude of a sphere, $S\left( q,D_{f},L \right)$ is the formulation from scattering fractals proposed by Teixeira^[48]^, and $S_{P}\left( q,r_{S}+r_{P} \right)$ the sin(x)/x term as in the Debye equation^[64]^. For the traces compared in Figure S7 2, L = 2 nm. When simulating the Pt_3_Co catalyst: R_P_ = 0.7 nm, σ_P_ = 0.25 nm, D_f_ = 2.5, L = 9 nm. The scattering contrast was calculated as^[25,69]^ $\Delta\rho={z\bar{\rho}}/{m_{M}}$, where z is the number of electrons of the specimen, $\bar{\rho}$ its density (in g cm^-3^), m_M_ is the molar mass. For the support, made of carbon: $z_{C}=6$, $\bar{\rho}_{C}=2 g cm^{-3}$, and $m_{M,C}=12 g mol^{-1}$, leading to ${\Delta\rho}_{S}=1 F mol^{-1}$. When the catalyst was made by bare Pt: $z_{Pt}=78$, $\bar{\rho}_{C}=21.45 g cm^{-3}$, $m_{M,C}=195 g mol^{-1}$, and ${\Delta\rho}_{P}=8.6 F mol^{-1}$. For Pt_3_Co:${\Delta\rho}_{P}=\left( 3{\Delta\rho}_{Pt}+{\Delta\rho}_{Co} \right)/4$, where ${\Delta\rho}_{Co}=4.1 F mol^{-1}$was retrieved with $z_{Co}=27$, $\bar{\rho}_{C}=8.9 g cm^{-3}$, and $m_{M,C}=59 g mol^{-1}$. Finally, according to the aforementioned works^[25,69]^: $N_{P}={\phi_{P}V_{avb}}/{V_{P}}={\phi_{P}\left[ \left( R_{S}+R_{P} \right)^{3}-R_{S}^{3} \right]}/{R_{P}^{3}}$, where $V_{P}$ is the particle volume, $V_{avb}$ the volume available in which decorating particles can be dispersed, and $\phi_{P}$the particle volume fraction. This latter amount can be estimated as $\phi_{P}={C\phi_{S}\bar{\rho}_{C}}/{\bar{\rho}_{Cat}}$, where C is the catalyst load (percentage catalyst mass with respect to support mass, equal to 0.2 in case of Pt and 0.3 in case of Pt_3_Co), $\phi_{S}$ is the support volume fraction (set at the maximum value for Vulcan XT72, 0.48^[69]^). With Pt catalyst: $N_{P}\approx120$, with Pt_3_Co: $N_{P}\approx200$.

In Figure S7, the evolution of $I_{tot}$is compared with the same pattern calculated without including the cross-correlation term ($I_{{tot,I}_{SP}=0}$). To better highlight the differences, on top of the graphs, the ratio among the two curves, $I_{r}={I_{tot,I_{SP}=0}}/{I_{tot}}$, is shown. As a result, the divergence from the scattering pattern calculated with the sum of all contributions can be quantified to be equal to 5% at its maximum. Such a deviation was considered acceptable of the benefits connected to neglecting its contribution and adopting the Schultz distribution, such as (i) simplification of modeling the CCM and (ii) lightening the computational load due to the replacement of the numerical integration with an analytical one.


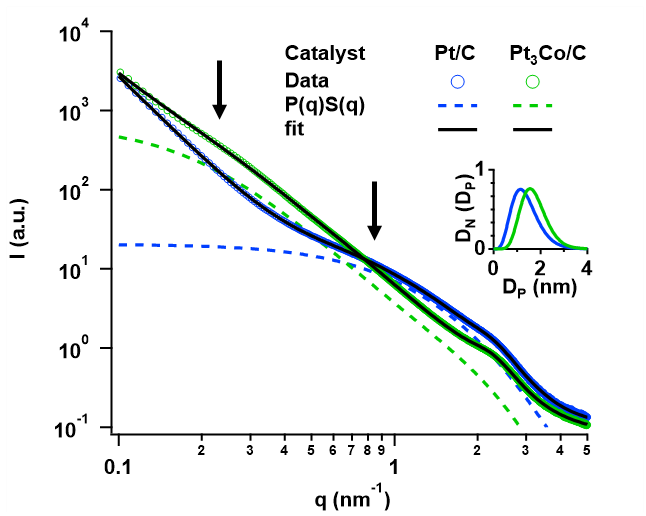


**Figure S4. SAXS characterization in pristine conditions.** CCMs loaded with Pt and Pt_3_Co catalyst nanoparticles, in pristine conditions are compared. Black lines represent the fit of the measured data carried out with the proposed analytical model. Dashed lines represent the product by the form and the structure factor used to model the scattering nanoparticles. The black arrows highlight the shoulder formed by the structure factor. Particle size distributions for the two samples, calculated from fit results, are displayed in the insert.

**Figure S5. Main SAXS results for scan in the middle position.** Main parameters obtained by fitting the scattering patterns with the analytical model along the vertical direction (as highlighted in figures S5 and S6): mean particle size (D_P_) and standard deviation (σ_P_) within the Schulz distribution, fractal dimension (D_f_), and fractal cut-off distance (L), related to average size of clustered nanoparticles. Results are displayed for all of the aged samples measured. Shaded areas highlight the extension along the vertical direction of the ROI analysed in proximity of the gas inlets (blue) and outlets (green) of the fuel cell.


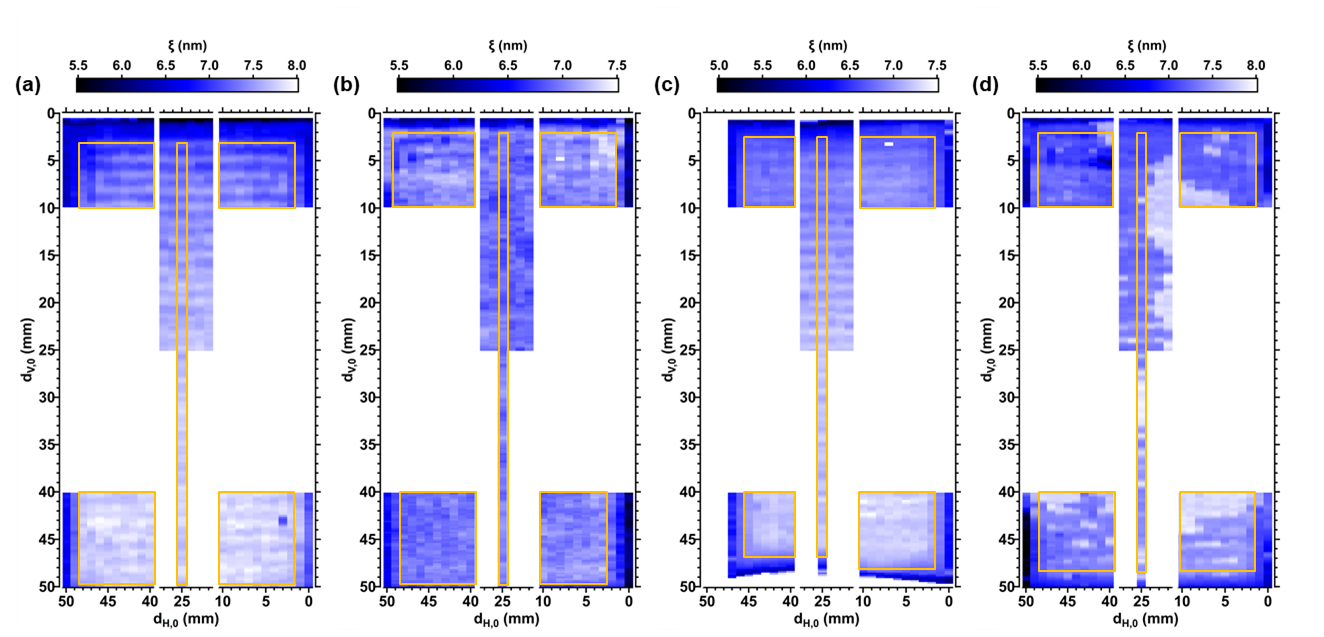


**Figure S6. SAXS correlation length maps.** From the scattering patterns collected from samples (a) Pt/AST1, (b) Pt/AST2, (c) Pt_3_Co/AST1, and (d) Pt_3_Co/AST2, scattering correlation length (ξ) was calculated within the q-range 0.157 ÷ 2.027nm^-1^. The areas delimited by the yellow frames define the points further subjected to quantitative analysis by least square fitting.


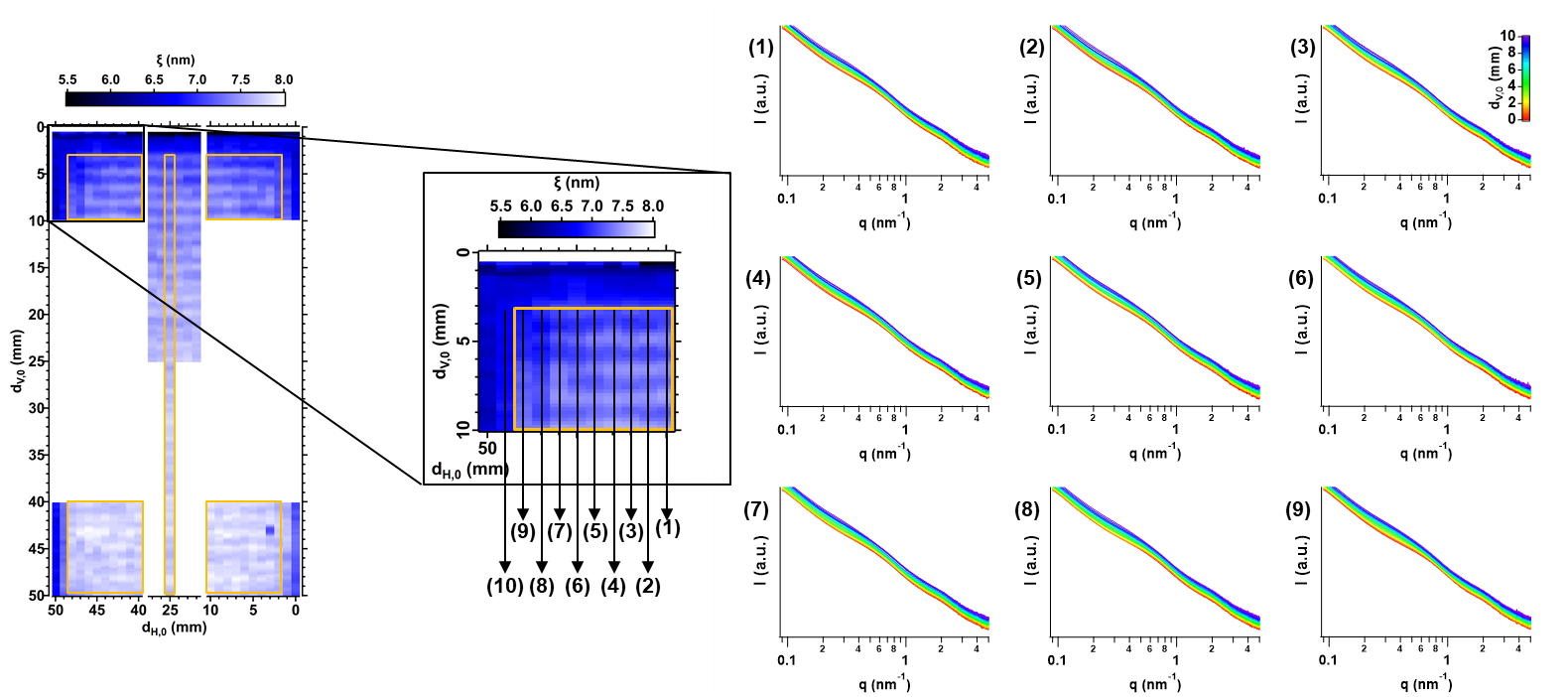


**Figure S7. SAXS data fitting.** Example of methodology used for fitting SAXS patterns on the Pt/C AST1 CCM. Once defined a Region Of Interest (ROI) within a correlation length map, traces were singularly fitted following a vertical scan (scan line (10) was not used when analysing this exemplative ROI, but it was used for other ROI). Results from all of the CCMs analysed are appended in Figures S8 to S11.

**Figure S8. Evaluation of the impact the cross-correlation term.** The overall scattering pattern of a support particle decorated with catalyst nanoparticles (I_tot_) is calculated for the two catalyst compositions as the sum of: (i) a term describing the particle support, I_S_, (ii) a term modelling the nanoparticles as a product by a form factor and a structure factor, I_PP_, and (iii) a term describing the cross-correlation term among these two subjects, I_SP_. I_tot_ is here compared with the three deconvoluted terms composing it and the same trace calculated in the cross-correlation term is neglected ($I_{{tot,I}_{SP}=0}$). On top, the difference is highlighted in terms of the ratio: $I_{r}={I_{{tot,I}_{SP}=0}}/{I_{tot}}$.


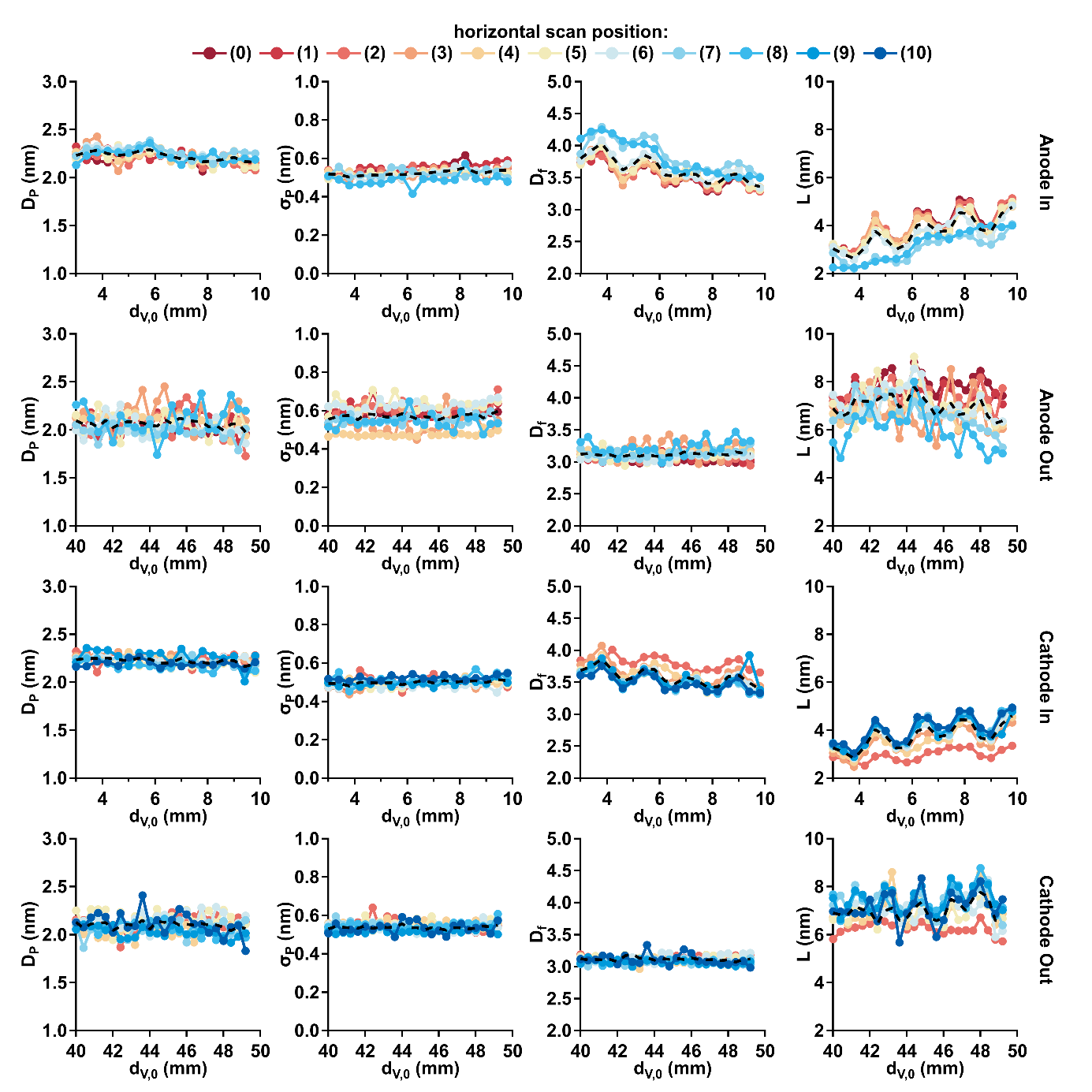


**Figure S9. Main SAXS results for Pt/C CCM after AST1.** Main parameters obtained by fitting the scattering patterns with the analytical model: mean particle size (D_P_) and standard deviation (σ_P_) within the Schulz distribution, fractal dimension (D_f_), and fractal cut-off distance (L), related to the average size of clustered nanoparticles. Dashed black lines represent the average value calculated per each position along the horizontal scan direction.


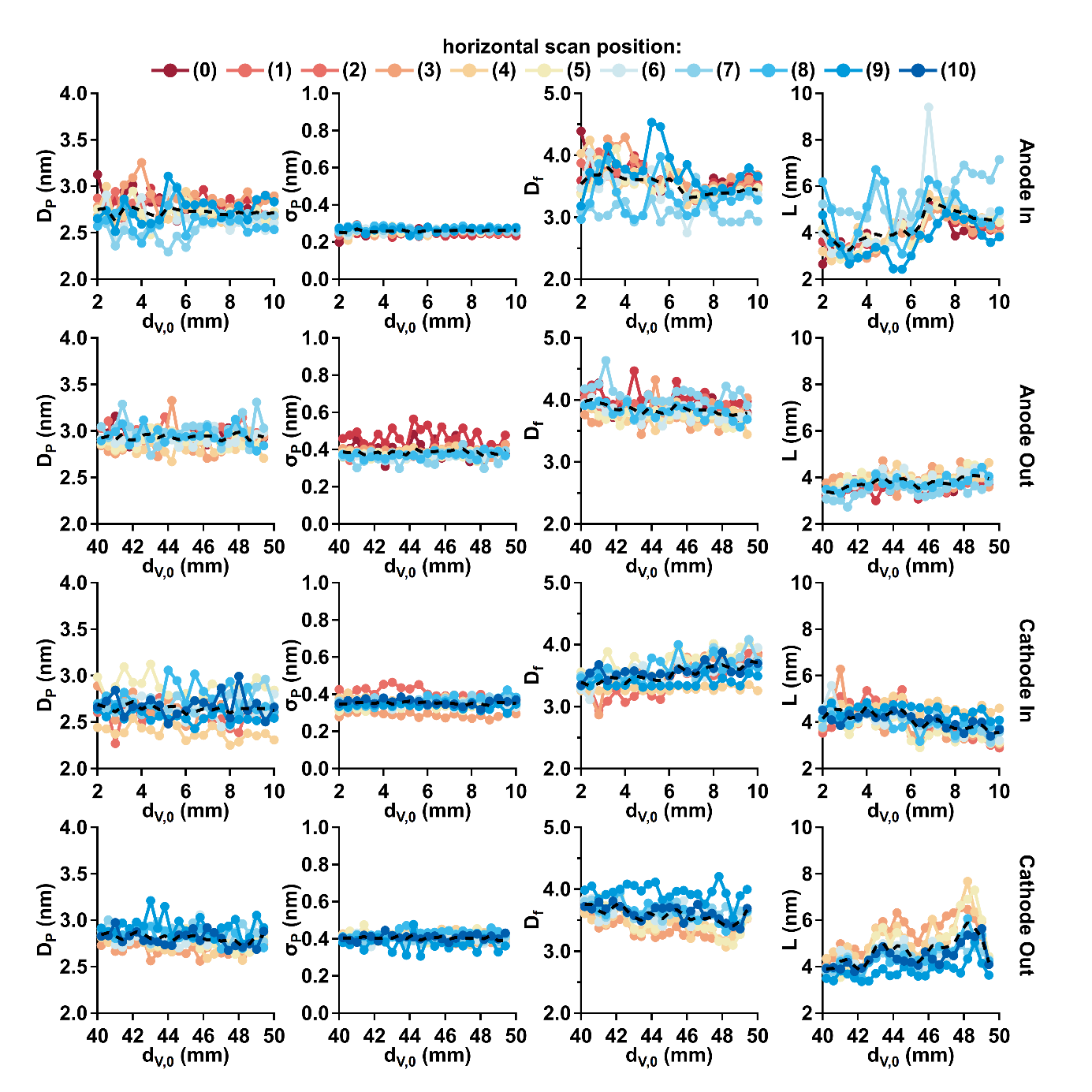


**Figure S10. Main SAXS results for Pt/C CCM after AST2.** Main parameters obtained by fitting the scattering patterns with the analytical model: mean particle size (D_P_) and standard deviation (σ_P_) within the Schulz distribution, fractal dimension (D_f_), and fractal cut-off distance (L), related to the average size of clustered nanoparticles. Dashed black lines represent the average value calculated per each position along the horizontal scan direction.


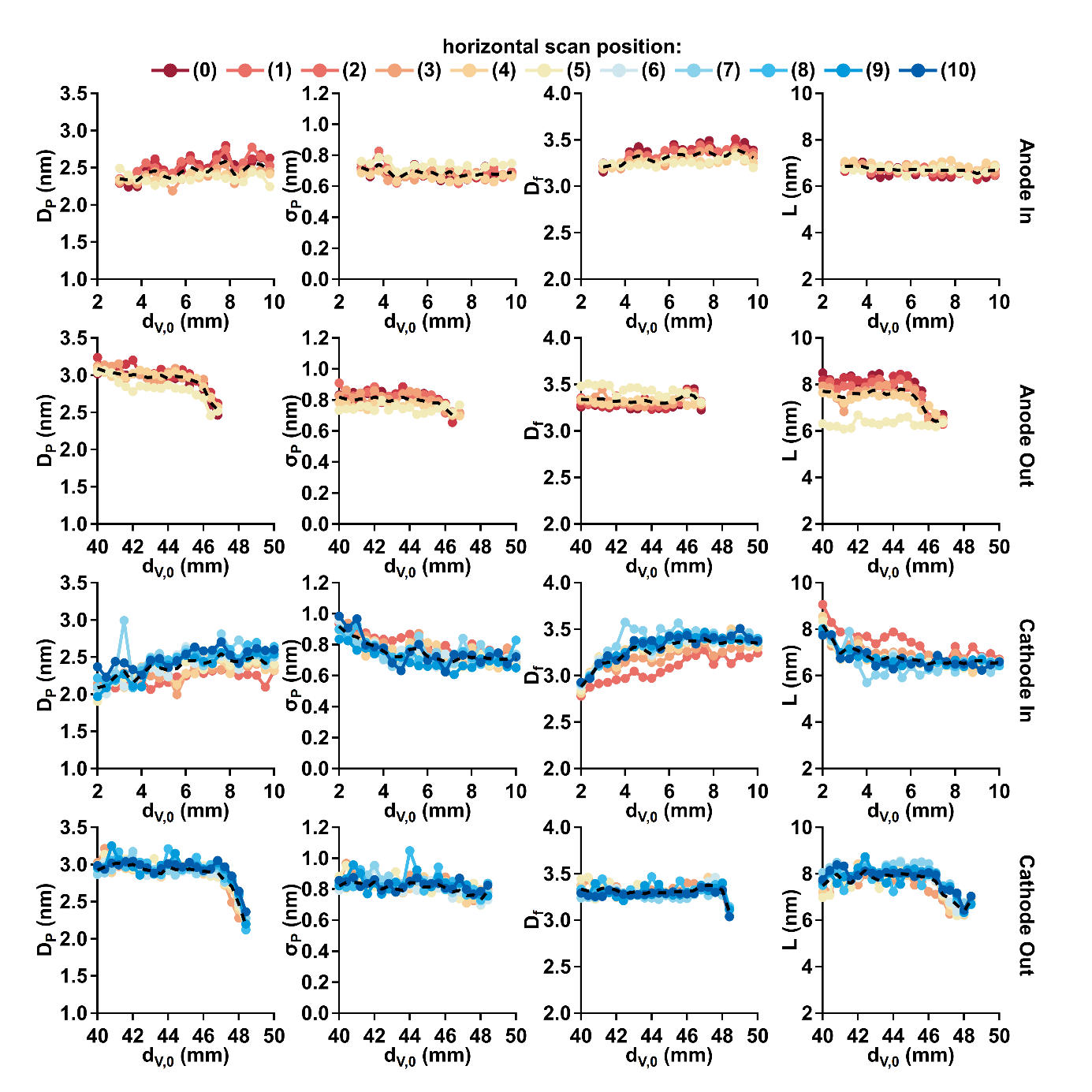


**Figure S11. Main SAXS results for Pt_3_Co/C CCM after AST1.** Main parameters obtained by fitting the scattering patterns with the analytical model: mean particle size (D_P_) and standard deviation (σ_P_) within the Schulz distribution, fractal dimension (D_f_), and fractal cut-off distance (L), related to average size of clustered nanoparticles. Dashed black lines represent the average value calculated per each position along the horizontal scan direction.


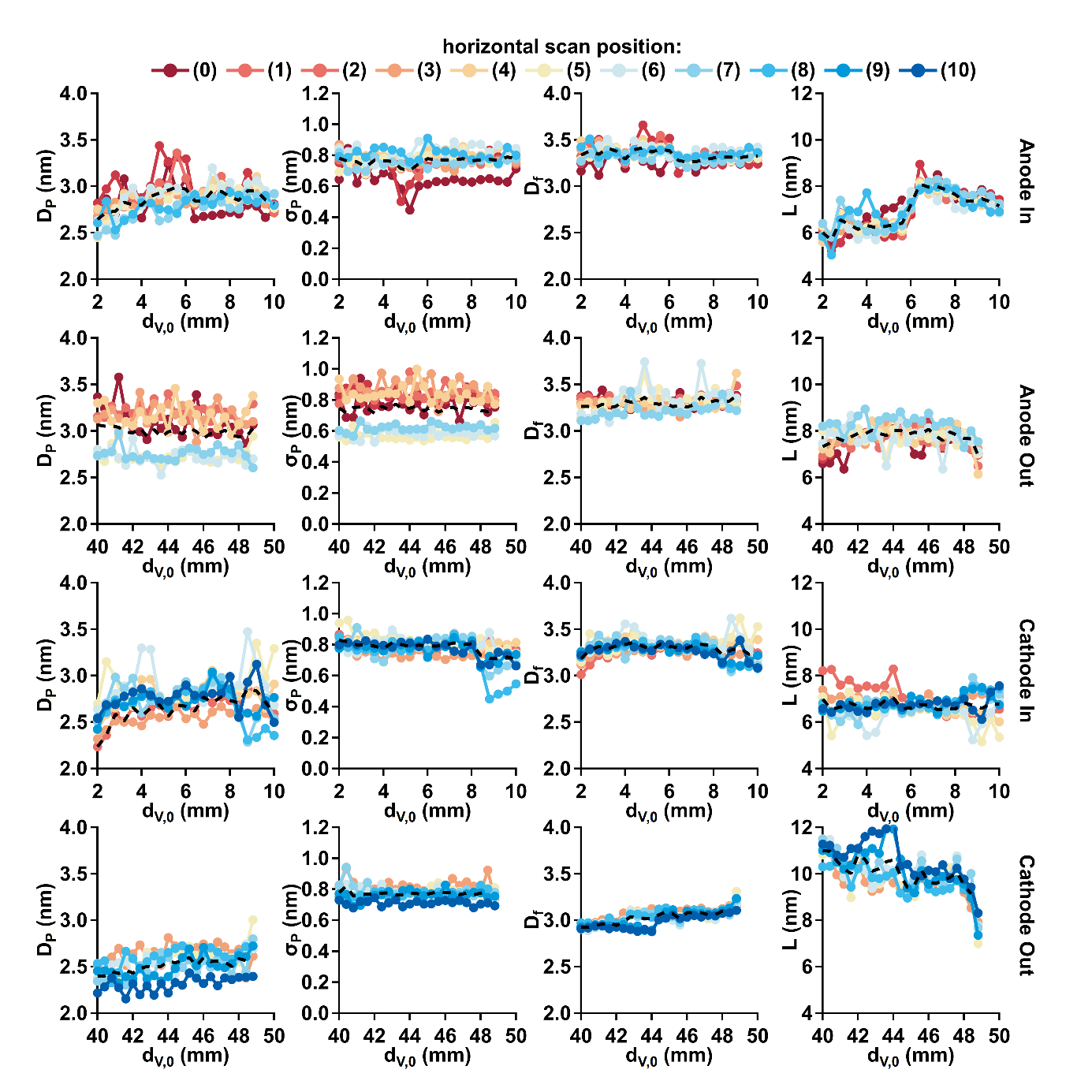


**Figure S12. Main SAXS results for Pt_3_Co/C CCM after AST2.** Main parameters obtained by fitting the scattering patterns with the analytical model: mean particle size (D_P_) and standard deviation (σ_P_) within the Schulz distribution, fractal dimension (D_f_), and fractal cut-off distance (L), related to the average size of clustered nanoparticles. Dashed black lines represent the average value calculated per each position along the horizontal scan direction.

**Table S1.** Comparison of the main parameters resulting from least square fitting EIS measurements by means of the equivalent circuit model**:** the charge transfer resistance (R_ct,_), the mass transport resistance (R_mt_), the membrane electrolyte resistance (R_el_), and the resistance of proton transport in the catalyst layer (R_pt_).

|  | Pt-based/ AST1(mΩ cm²) | | Pt-based/AST2(mΩ cm²) | | Pt_3_Co-based/AST1(mΩ cm²) | | Pt_3_Co-based/AST2(mΩ cm²) | |
| --- | --- | --- | --- | --- | --- | --- | --- | --- |
|  | BoT | EoT | BoT | EoT | BoT | EoT | BoT | EoT |
| R_ct_ | 418.75 | 353.32 | 497 | 489.25 | 585 | 679.75 | 678.25 | 2349.5 |
| R_mt_ | 130 | 126.75 | 187.5 | 212 | 226 | 241 | 298 | 953.5 |
| R_el_ | 89.75 | 92 | 89.5 | 94 | 87.25 | 90.25 | 83 | 82.5 |
| R_pt_ | 21 | 24.5 | 23.75 | 34.75 | 50 | 44.25 | 33 | 26.75 |

**Table S2. Pt-based CCM: fit results.** Comparison of the main parameters resulting from least square fitting the measured scattering patterns with the presented analytical model: mean particle size (D_P_) and standard deviation (σ_P_) within the Schulz distribution, fractal dimension (D_f_), and cut-off distance (L), related to average cluster size.

| Status | D_p_ (nm) | σ_p_ (nm) | D_f_ | L (nm) |
| --- | --- | --- | --- | --- |
| Pristine | 1.382 ± 0.039 | 0.573 ± 0.020 | 2.191 ± 0.207 | 2.054 ± 0.221 |
| AST1 |  |  |  |  |
| Anode Inlet (162) | 2.222 ± 0.007 | 0.524 ± 0.027 | 3.632 ± 0.233 | 3.686 ± 0.738 |
| Anode Outlet (216) | 2.064 ± 0.118 | 0.572 ± 0.057 | 3.115 ± 0.097 | 6.988 ± 0.798 |
| Cathode Inlet (162) | 2.223 ± 0.059 | 0.500 ± 0.023 | 3.586 ± 0.165 | 3.746 ± 0.616 |
| Cathode Outlet (216) | 2.089 ± 0.094 | 0.541 ± 0.023 | 3.100 ± 0.057 | 7.089 ± 0.631 |
| AST2 |  |  |  |  |
| Anode Inlet (189) | 2.718 ± 0.149 | 0.259 ± 0.015 | 3.501 ± 0.293 | 4.350 ± 0.985 |
| Anode Outlet (216) | 2.936 ± 0.114 | 0.386 ± 0.041 | 3.848 ± 0.183 | 3.768 ± 0.336 |
| Cathode Inlet (189) | 2.648 ± 0.167 | 0.352 ± 0.036 | 3.528 ± 0.218 | 4.087 ± 0.562 |
| Cathode Outlet (192) | 2.810 ± 0.107 | 0.405 ± 0.028 | 3.589 ± 0.224 | 4.664 ± 0.818 |

**Table S3. Pt_3_Co-based CCM: fit results.** Comparison of the main parameters resulting from least square fitting the measured scattering patterns with the presented analytical model: mean particle size (D_P_) and standard deviation (σ_P_) within the Schulz distribution, fractal dimension (D_f_), and cut-off distance (L), related to average cluster size.

| Status | D_p_ (nm) | σ_p_ (nm) | D_f_ | L (nm) |
| --- | --- | --- | --- | --- |
| Pristine | 1.371 ± 0.023 | 0.549 ± 0.004 | 2.670 ± 0.022 | 9.943 ± 0.528 |
| AST1 |  |  |  |  |
| Anode Inlet (108) | 2.415 ± 0.106 | 0.691 ± 0.436 | 3.289 ± 0.633 | 6.686 ± 0.757 |
| Anode Outlet (108) | 2.938 ± 0.161 | 0.780 ± 0.049 | 3.347 ± 0.066 | 7.186 ± 0.681 |
| Cathode Inlet (126) | 2.364 ± 0.180 | 0.573 ± 0.077 | 3.267 ± 0.166 | 6.830 ± 0.512 |
| Cathode Outlet (198) | 2.866 ± 0.220 | 0.809 ± 0.067 | 3.310 ± 0.079 | 7.599 ± 0.636 |
| AST2 |  |  |  |  |
| Anode Inlet (189) | 2.865 ± 0.157 | 0.762 ± 0.070 | 3.337 ± 0.083 | 6.943 ± 0.801 |
| Anode Outlet (184) | 2.991 ± 0.230 | 0.742 ± 0.131 | 3.230 ± 0.094 | 7.762 ± 0.490 |
| Cathode Inlet (189) | 2.741 ± 0.192 | 0.781 ± 0.069 | 3.295 ± 0.096 | 6.713 ± 0.487 |
| Cathode Outlet (198) | 2.563 ± 0.399 | 0.905 ± 0.412 | 3.051 ± 0.237 | 10.007 ± 0.866 |

**Table S4. ICP-MS analysis of cathode water produced while running AST2 on Pt_3_Co-based MEA.**

| Element | Pt | Pt_3_Co |
| --- | --- | --- |
| Concentration in effluent water (µgL^-1^) | - | 0.28 ± 0.01 |
